# Supplementary material for: d-Allulose Improves Endurance and Recovery from Exhaustion in Male C57BL/6J Mice
Source: Nutrients. 2022 Jan 18;14(3):404. doi: 10.3390/nu14030404 (PMC8838150; doi:10.3390/nu14030404)
Supplement: Supplementary file 1 [file nutrients-14-00404-s001.zip › nutrients-1546200-supplementary.pdf]

Supplementary Materials

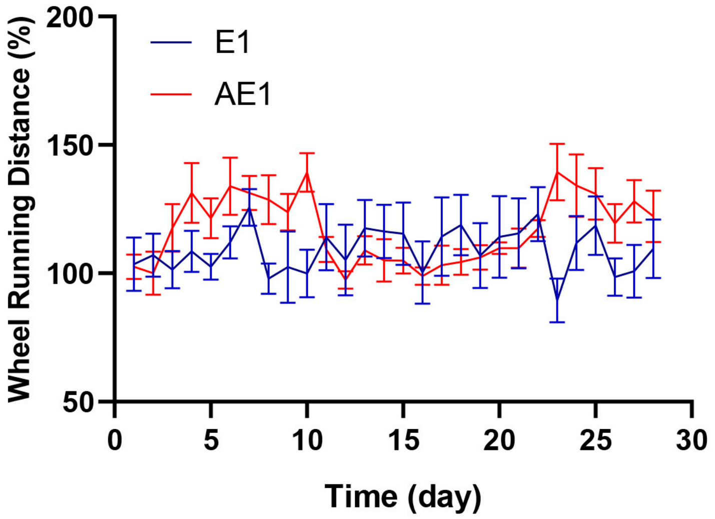

Figure S1. Changes in running distance during the 4 weeks. The daily distance is the percent of the baseline activity. E1: exercise group fed with chow diet; AE1: exercise group fed with D-allulose diet.

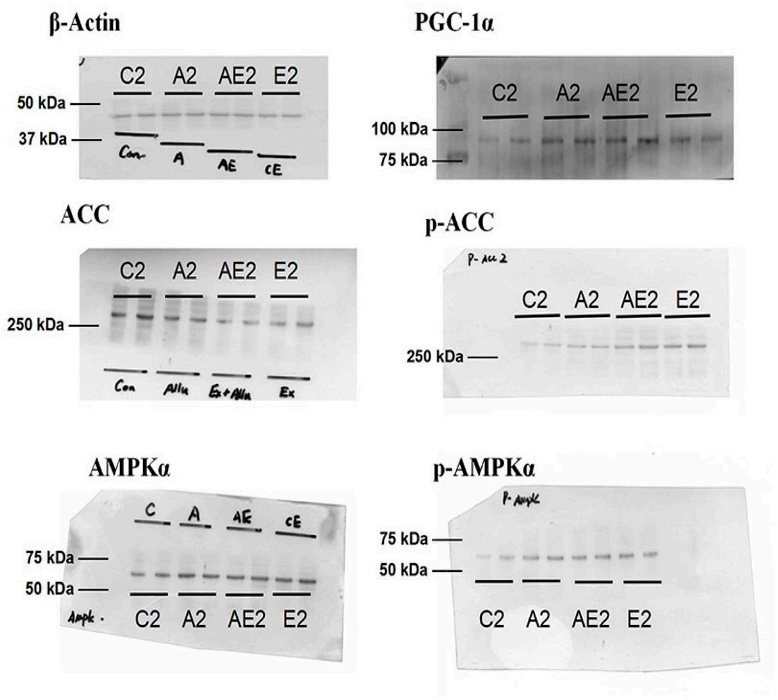

Figure S2. Uncropped images of Western blots. AMPK: AMP-activated protein kinase; p-AMPK: phosphorylated AMPK; ACC: acetyl-CoA carboxylase; p-ACC: phosphorylated ACC; PGC-1α: peroxisome proliferator-activated receptor γ coactivator 1α. C2: sedentary group fed with chow diet; A2: sedentary group fed with D-allulose diet; E2: exercise group fed with chow diet; AE2: exercise group fed with D-allulose diet.

Table S1. Composition of the experimental diets.

Ingredients	Chow diet (%)	D-allullose diet (%)
Cornstarch	38.6	38.6
Casein	19.4	19.4
pregelatinized Cornstarch	12.8	12.8
Granulated sugar	9.7	9.7
Soybean oil	6.8	6.8
Cellulose	7.9	4.9
D-allulose	0.0	3.0
Mineral mixture	3.4	3.4
Vitamin mixture	1.0	1.0
L-cystine	0.3	0.3
Choline bitartrate	0.2	0.2
t-Butylhydroquinone	0.001	0.001
Total	100	100
